# Current Implications of microRNAs in Genome Stability and Stress Responses of Ovarian Cancer

**DOI:** 10.3390/cancers13112690

**Published:** 2021-05-29

**Authors:** Arkadiusz Gajek, Patrycja Gralewska, Agnieszka Marczak, Aneta Rogalska

**Affiliations:** Department of Medical Biophysics, Faculty of Biology and Environmental Protection, Institute of Biophysics, University of Lodz, Pomorska 141/143, 90-236 Lodz, Poland; arkadiusz.gajek@biol.uni.lodz.pl (A.G.); patrycja.gralewska@edu.uni.lodz.pl (P.G.); agnieszka.marczak@biol.uni.lodz.pl (A.M.)

**Keywords:** microRNA, ovarian cancer, PARP, replication stress, targeted therapy

## Abstract

**Simple Summary:**

Ovarian cancer is the leading cause of death from gynecological malignancies. Recent studies have focused on ovarian cancer-associated microRNAs that play strong regulatory roles in various cellular processes. While miRNAs have been shown to participate in regulation of tumorigenesis and drug responses through modulating the DNA damage response (DDR), little is known about their potential influence on sensitivity to chemotherapy. The main objective of this review is to summarize recent findings on the utility of miRNAs as ovarian cancer biomarkers and their regulation of DDR or modified replication stress response proteins.

**Abstract:**

Genomic alterations and aberrant DNA damage signaling are hallmarks of ovarian cancer (OC), the leading cause of mortality among gynecological cancers worldwide. Owing to the lack of specific symptoms and late-stage diagnosis, survival chances of patients are significantly reduced. Poly (ADP-ribose) polymerase (PARP) inhibitors and replication stress response inhibitors present attractive therapeutic strategies for OC. Recent research has focused on ovarian cancer-associated microRNAs (miRNAs) that play significant regulatory roles in various cellular processes. While miRNAs have been shown to participate in regulation of tumorigenesis and drug responses through modulating the DNA damage response (DDR), little is known about their potential influence on sensitivity to chemotherapy. The main objective of this review is to summarize recent findings on the utility of miRNAs as cancer biomarkers, in particular, ovarian cancer, and their regulation of DDR or modified replication stress response proteins. We further discuss the suppressive and promotional effects of various miRNAs on ovarian cancer and their participation in cell cycle disturbance, response to DNA damage, and therapeutic functions in multiple cancer types, with particular focus on ovarian cancer. Improved understanding of the mechanisms by which miRNAs regulate drug resistance should facilitate the development of effective combination therapies for ovarian cancer.

## 1. Introduction

Ovarian cancer is the leading cause of death from gynecological malignancies. The high fatality rate is linked to the complexity of the disease and consequent difficulty in making an accurate diagnosis. At the initial stages of disease progression, patients present with non-specific symptoms [1]. The majority of cases are diagnosed at the third or fourth stage of clinical advancement following the spread of disease to other organs. At present, standard treatment for ovarian cancer involves total removal of tumor mass and any tissue that may pose risk of spread. In the case of highly advanced tumors, radical surgery is not possible [2]. Debulking surgery is performed, followed by adjuvant treatment with drugs containing platinum compounds or taxane-based chemotherapy, which has shown success in improving the survival rates of patients in the fourth stage of the disease. However, the five-year survival rate for advanced-stage cases remains below 30% [3]. Ovarian cancer cells often carry BRCA1 or BRCA2 (BRCA1/2), germline or somatic mutations in ataxia telangiectasia and Rad3-related protein (ATR) or checkpoint kinase 1 (CHK1) genes of the homologous recombination (HR) pathway [4]. Poly (ADP ribose) polymerase inhibitors (PARPis) have been identified as the most promising targeted therapy for ovarian cancer. In the United States, the FDA approved olaparib for maintenance treatment of patients with BRCA^MUT^ advanced epithelial ovarian cancer (EOC) showing complete or partial response to first-line platinum-based chemotherapy in 2018 [5].

Elucidation of molecular alterations in serous ovarian carcinoma cells is necessary to identify novel targets for early detection and treatment. Recent studies have focused on ovarian cancer-associated microRNAs (miRNAs) that play strong regulatory roles in various cellular processes. Initial findings support a potential correlation between miRNAs and cancer development. Even low-level disruption in expression patterns of individual miRNAs can lead to significant pathological changes, such as neoplasia. Alterations in miRNA expression are widely reported in multiple cancer types, especially ovarian cancer resistant to chemotherapy [6]. Knowledge of the specific associations between miRNAs and DNA damage response (DDR) or DNA repair should aid in expanding applications of miRNAs in cancer therapy. Cells detect DNA damage and coordinate an appropriate response involving activation of repair pathways, such as nucleotide excision repair (NER) and HR. If damage is too excessive for repair, an apoptotic response is initiated through activating death receptors or triggering intrinsic apoptosis. 

In this review, earlier findings on the direct effects of miRNAs on sensitivity of ovarian cancer cells to replication stress response (RSR) inhibitors are described. The therapeutic potential of the miRNAs regulating DDR/DNA repair is discussed, along with the mechanisms by which miRNAs affect sensitivity to PARP, ATR, and CHK1 inhibitor therapy. The identification of mediator miRNAs that improve response to treatment with checkpoint inhibitors would increase the proportion of patients benefiting from therapy.

## 2. Participation of miRNAs in Pathogenesis and Development of Neoplastic Diseases

**Figure 1:**
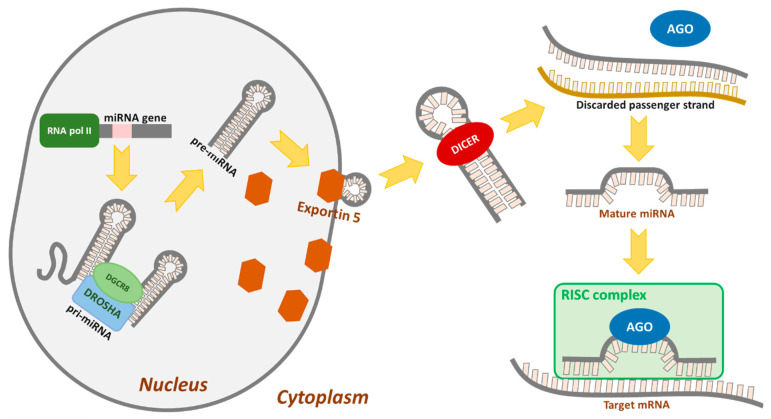
The main molecular steps involved in miRNA formation and organization.

MiRNAs are a class of small, endogenous, and noncoding RNAs that post-transcriptionally regulate gene expression. MiRNA is transcripted in the nucleus, usually by RNA polymerase II, to produce the primary miRNA (pri-miRNA). Pri-miRNA is identified and cleaved by the Drosha, an RNase III enzyme, and its cofactor DGCR8 (Pasha), which form a hairpin precursor miRNA (pre-mRNA). The hairpin precursor is exported out of the nucleus by Exportin 5, where Dicer (RNase III enzyme) cleaves double stranded mi-RNA and creates miRNA as transient 21–24 nucleotide duplex miRNA. The strand of mature sequence is then transported onto Argonaute (Ago) and is loaded into a protein complex called RISC. MiRNA recognizes their target sequences based on complementarity to the 3′untranslated region (3′UTR) of mRNA transcripts, leading to translational inhibition and/or mRNA degradation [7,8], Figure 1. 

Almost half the miRNA genes are located in fragile sites of the genome where chromosome fragments are lost or rearranged with high frequency. Mutations in these areas are often linked to cancer development, implicating microRNAs in the formation and progression of neoplasms [7,8,9]. Expression of miRNA genes located near these regions is commonly disrupted. One example is miR-15a and miR-16-1 genes located on the long arm of chromosome 13 in region 14.2 where deletions are frequent. Reduced levels or complete absence of miR-15a and miR-16-1 are detected in many patients with B-cell chronic lymphocytic leukemia, ovarian and prostate cancer, mantle cell lymphoma, and multiple myeloma [10,11,12]. MiRNAs are also secreted from both normal and cancer cells in exosomes, small vesicles that play a key role in cell-to-cell communication in the body [13]. In addition, variable environmental factors, such as low pH and hypoxia (characteristic of most solid tumors, including ovarian cancer), affect miRNA expression and promote exosome secretion. Several indications support the potential utility of miRNAs circulating in the bloodstream as biomarkers of cancer. One hypothesis is that miRNAs appear in the bloodstream through two mechanisms, one associated with tissue damage, as demonstrated in earlier studies (e.g., miR-208 is observed in serum after cardiac muscle damage) [14], and the second related to so-called microbubbles (exosomes) involved in tumor-associated immunosuppression, metastasis, and angiogenesis that are derived directly from the cytoplasmic membrane and reflect the antigenic composition of parent cells [15]. Secreted miRNAs can also be an additional source of information about defects in the DNA repair system, including those related to replication stress (miR-200c, miR-214 [16], miR-185-5p [17], miR-126, miR-17, miR-92a [18], and miR-34a [19]). In 2008, Taylor et al. first demonstrated that eight exosomal miRNAs (miR-21, miR-141, miR-200a, miR-200b, miR-200c, miR-203, miR-205, and miR-214) were elevated in the serum of ovarian cancer patients, even in the case of patients with early stages of the disease. Very importantly, the miRNA profiles observed in exosomes were similar to those in the originating tumor cells. Circulating miRNA profiles accurately reflect the tumor profiles, which make them potential biomarkers and relevant for ovarian cancer diagnosis prognosis and therapeutics [16,20,21]. Since that time, other studies confirmed other circulating miRNA profiles in plasma samples of ovarian cancer as possible biomarkers of which some miRNA were significantly increased, e.g., hsa-miR-106a-5p, hsa-let-7d-5p, hsa-miR-93-5p [17], miR-1274a, miR-625-3p, and miR-720 [18], and others significantly decreased, e.g., hsa-miR-122-5p, hsa-miR-185-5p and hsa-miR-99b-5p [17], miR-106b, miR-126, miR-150, miR-17, miR-20a, and miR-92a [18]. Maeda et al. recently described the potential role of serum miR-34a in early diagnosis of ovarian cancer and for histological subtyping of EOC [19]. In the case of ovarian cancer patients, the elevated level of miRNA (in comparison to healthy) was reported not only in serum exosomes, but also, e.g., in ascites: miR-21, miR-23b, or miR-29a [22], or in urine: miR-30-5p [23]. It is considered that these exosomes are responsible for inducing more aggressive disease, so it confirms that they also might serve as a promising diagnostic and therapeutic targets [21,22]. The correlation between the increased levels of miR-200b and miR-200c with the main marker of ovarian cancer-CA125, commonly used in diagnosis [24], was also observed. Additionally, studies performed by Kapetanakis et al. [25] demonstrated that miR-200b was able to predict the sensitivity to treatment in much more sensitive manner than CA125. After primary treatment (surgery and chemotherapy) of the group of 33 ovarian cancer patients, CA125 very quickly (even after 1 month after treatment) returned to a normal level in almost all patients, whereas there was a difference in the level of miR-200b between individuals. The patients with a negative miR-200b variation had a longer progression-free survival (PFS), than those patients with a positive variation. Increased levels of specific mRNAs characteristic of certain cancer types (breast, lung, ovary, prostate, pancreas, liver, and colon cancer and chronic myeloid leukemia) are often associated with tumor invasiveness or metastasis. The miRNA molecules known to inhibit the processes of migration and invasion of neoplastic cells include miR-149 (breast cancer), miR-138 (ovarian and kidney cancer), miR-126 (lung and stomach cancer), and miR-206 (melanoma and cervical cancer), among others. Additionally, miR-373 and miR-520c are associated with the invasive and metastatic ability of cancer cells. These molecules directly inhibit expression of the CD44 surface receptor responsible for binding hyaluronan (the main component of the extracellular matrix), an intermediary for several stimulatory processes, such as migration and proliferation. Moreover, miRNAs are involved in epithelial-to-mesenchymal transition (EMT), a necessary step in metastasis. Increased levels of specific miRNAs are additionally associated with the occurrence of epigenetic abnormalities in cancer cells [26,27] Molecular functions of miRNAs in ovarian cancer acting as oncogenes or suppressors are presented in Table 1. 

## 3. Aberrant Expression Profiles of miRNAs in Ovarian Cancer

Recent studies revealed differences in the miRNA expression profiles in tissues from patients with ovarian cancer and healthy individuals. Increased miRNA levels were not detected in patients with benign ovarian disease [73]. Further comparison of expression levels of miRNAs in ovarian cancer revealed distinct roles of different miRNAs. Expression of miR-200a, miR-200b, and miR-200c was significantly higher than that in normal tissues, whereas mir-199a, miR-140, miR-145, and miR-125b1 displayed low expression in ovarian cancer tissues [74]. MiR-10a and miR-10b suppressed proliferation of granulosa cells in the ovary. The miR-10 family suppressed expression of several key genes in the transforming growth factor beta (TGF-β) pathway, suggesting a negative feedback loop between the miR-10 family and TGF-β pathway [34]. Numerous studies performed on various tissue types have validated the utility of miRNAs as a prognostic marker of ovarian cancer [75,76,77].

### 3.1. MiRNAs as Tumor Suppressors

Suppressor genes, also known as anti-oncogenes, encode proteins that inhibit the processes of cell growth and differentiation and maintain genetic stability of the cell. Mutations in these genes can lead to uncontrolled cell proliferation and, consequently, development of cancer. The effect of miRNAs in the case of their activation or deactivation will lead to an insufficient number of target genes or their overexpression, respectively. Target miRNA transcripts determine whether the miRNA should be considered an oncogene or a tumor suppressor [78,79]. Biological functions of miRNAs depend on the cellular context, tumor molecular subtype, stage of tumor progression, or interactions with therapy [80]. It was observed that miR-200c and miR-141 produce resistance to carboplatin while sensitizing MES-OV/TP cells to paclitaxel. The authors suggest that the effects of these miRNAs on drug sensitivity are cell context dependent [6]. Higher miR-200c levels were also associated with better progression-free survival in stage I epithelial ovarian cancer [81]. Liu et al. found that miR-200b and miR-200c increased cisplatin sensitivity through downregulation of DNA methyltransferases (DNMT3A/DNMT3B) and the indirect downregulation of DNMT1 by targeting Sp1 transcription factor [26]. Based on 220 ovarian cancer patients’ analysis, it was observed that overexpression of miR-200c correlated with poor or good outcome depending on the cellular localization of HuR (RNA binding protein). MiR-200c can act either as a suppressor or enhancer of the aggressive phenotype, depending upon the localization of HuR. Suppressor genes contribute to drug resistance of several types of solid tumors [82]. Tumor suppressor miRNAs prevent tumor development through negative regulation of genes that control cell differentiation or apoptosis. To date, a number of miRNAs have been identified as tumor suppressors [83]. For instance, miR-29 significantly reduces migration of highly metastatic ovarian cancer cells [36]. Expression of miR-29 alone or in combination with cisplatin could effectively reduce tumorigenicity of CP70 ovarian cancer cells [35]. 

One of the most well-characterized tumor suppressors of the miRNA family in ovarian cancer is Let-7, which belongs to a family of highly homologous members. Ten mature subtypes of the human let-7 family have been identified to date, whereby mature let-7a and let-7f are processed from precursor sequences (let-7a-1, let-7a-2, let-7a-3; let-7f-1, and let-7f-2) [38,39,40]. Let-7 inhibits cell proliferation and increases apoptosis by inhibiting expression of proto-oncogenes, such as the small GTPase RAS, high mobility group AT-hook 2 (HMGA 2), c-Myc, cell division cycle homolog 25A (CDC25A), cdk 6, and cyclin 2 [39,84]. Overexpression of let-7g miRNA in OVCAR3 and HEY-A8 EOC cells induced cell cycle arrest, slowed progression of EMT, and significantly improved cell response to cis-platinum treatment. Let-7g worked through vimentin and reduced the expression of Snail and Slug (the protein product of snail family transcriptional repressor 2) [39]. Other studies have demonstrated overexpression of miR-16 in ovarian cancer tissues, including SKOV3 and OVCAR3 cell lines, compared with normal ovarian epithelial cells. MiR-16 is reported to exert suppressive effects on cell migration and invasion by inactivation of the Wnt/β-catenin signaling pathway through decreasing expression of matrix metallopeptidase MMP2 and MMP9. Additionally, miR-16 regulates the expression of mesenchymal markers (cadherin 1 and 2, snail 1 and 2, vimentin, and twist family BHLH transcription factor) [28]. MiR-31 is another microRNA with biological significance. This miRNA is expressed at low levels in serous ovarian cancer cells and tissues and induces suppression of cell proliferation, clonogenic potential, and cell migration and invasion [43]. Recent research indicates that miR503HG interacts with and promotes methylation of miR-31-5p that play a role in inhibition of ovarian cancer cell invasion and migration [42]. MiR-506-3p inhibits proliferation and promotes apoptosis via inactivation of the NAD-dependent protein deacetylase sirtuin-1 (SIRT1)/AKT/Forkhead box protein 3a (FOXO3a) signaling pathway [45]. Myotubularin-related protein 6 (MTMR6) has been identified as another functional target of miR-506-3p. Several recent studies indicate that malignant biological behaviors are regulated by the myotubularin (MTM) protein family [85]. Other miRNAs acting as suppressors include miR-424-5p and miR-503-5p that directly target the 3′UTR of KIF23 (kinesin-6, a plus-end-directed motor protein in mitosis) to suppress its expression and inhibit ovarian cancer cell proliferation and migration [47]. Additionally, miR-199a-5p is reported to function as a suppressor of ovarian cancer (HO-8910 and ES-2) cell proliferation and invasion through inhibiting NF-κB1 expression. Notably, expression patterns of matrix metalloproteinases (MMP-2 and MMP-9) are altered in a similar manner as NF-κB1 upon exogenous expression of miR-199a-5p [51]. The anti-oncomiR list includes miRNAs from the miR-34 family that inhibit oncogenes, such as c-MYC and c-MET, or promote mitosis CDKs [53] and miR-340-5p. Deficiency of miR-340-5p promotes expression of serine/threonine-protein kinase B-raf (BRAF), NF-kB and ATP-binding cassette sub-family B member 5, also known as P-glycoprotein (ABCB5), resulting in development of drug resistance [55]. 

### 3.2. MiRNAs as Oncogenes

Alterations in expression of several miRNAs are observed in many cancer types [81,86,87]. Mutation in a single allele of proto-oncogenes can trigger transformation into oncogenes. These genes promote cancer development by negatively regulating the tumor genes responsible for cell differentiation or apoptosis [88]. Several miRNAs in tumor cells exhibit oncogenic traits and promote tumorigenesis. Notably, almost all members of the miR-200 family (miR-200a, miR-200b, miR-200c, miR-141, and miR-429) are upregulated in ovarian cancer [89]. Different miRNA types, including miR-182 and the miR-200 family (specifically, miR-200a, miR-200b, and miR-200c), are highly overexpressed in high-grade serous epithelial ovarian cancer (SEOC). The miR-200 family participates in EMT through regulating E-cadherin by inhibiting zinc-finger E-box-binding homeobox 1 (ZEB1) and zinc-finger E-box-binding homeobox 2 (ZEB2) [30] and improves response to paclitaxel (PTX) due to repression of the miR-200c target, ZEB1. The transcription factor, Grainyhead-like 2n (GRHL2), acts as a pivotal gatekeeper of EMT in EOC via miR-200-ZEB1 [31]. The miR-200 family also sensitizes ovarian cancer cells to PTX through downregulation of TUBB3/class III beta-tubulin, a component of microtubules that binds paclitaxel [90]. Moreover, in PTX resistant cells (A2780/1A9, MES-OV, OVCAR-3, ES-2), miR-200b and miR-200c were downregulated and associated with EMT, with increased vimentin, fibronectin1, MMP2, or MMP9 [90]. MiR-200a is reported to enhance sensitivity to PTX-induced reactive oxygen species production. Overexpression of miR-200a-3p markedly promotes proliferation, colony formation, and invasion of ovarian cancer cells. Expression of this miRNA in ovarian cancer tissues is significantly negatively correlated with that of Protocadherin-9, a potential tumor suppressor, in a variety of cancers [32]. Moreover, the miR-200 family plays a major role in regulating EMT and sensitivity to carboplatin and PTX in OVCAR-3 and MES-OV cells. Inhibition of miR-200c and miR-141 resulted in the downregulation of E-cadherin and the upregulation of vimentin and fibronectin [33]. 

MiR-205 expression is significantly increased with a simultaneous decrease in transcription factor 21 (TCF21, a tumor suppressor gene) in epithelial ovarian carcinoma compared to normal ovarian cells. Thus, miR-205 is regarded as an oncogene in ovarian cancer that plays critical roles in tumor invasion and metastasis [41]. MiRNA-126-3p is also implicated in cancer progression and inflammation. Overexpression of miR-126-3p in OVCAR3 cells is reported to suppress cell proliferation and invasion as well as phosphorylation of serine/threonine-specific protein kinase B (AKT) and extracellular signal-regulated kinases ½ (ERK1/2) [44]. MiR-183 exerts tumor-promoting effects in ovarian cancer by regulating one of the transcription factor proteins, Mothers against decapentaplegic homolog 4 (Smad 4), via the TGF-β/Smad4 pathway. MiR-183 is upregulated in OC tissues and cell lines. Downregulation of miR-183 via cell transfection inhibited proliferation and invasion and induced apoptosis in SKOV3 and OVCAR3 cells [46]. Expression of miR-760 is markedly upregulated in association with an aggressive phenotype of OC and poor prognosis [48,49]. Additionally, miR-151 plays an oncogenic role in carcinogenesis and progression of ovarian cancer by activating AKT/mTOR signaling through effects on the Rho guanine nucleotide dissociation inhibitor (RhoGDIA). MiR-151 activates Ras-related C3 botulinum toxin substrate 1 (Rac1), Cdc42, and Rho GTPase by directly targeting the 3-UTR of RhoGDIA, a metastasis suppressor [50]. Examples of oncomiRs include miR-21-5p, which controls the suppressor gene phosphatase and tensin homolog (PTEN, an inhibitor of the Akt kinase pathway) [52], miR-106a, which regulates the p21 protein level, and miR-195, which controls WEE1 kinase, an inhibitor of cell division [54]. MiR-222 is overexpressed in EOC cases and promotes proliferation through downregulation of target cyclin-dependent kinase inhibitor p27Kip1 [57]. An earlier study reported upregulation of miR-221 in 63 samples of ovarian cancer. A negative correlation between expression of apoptosis protease activator 1 (APAF1) protein and miR-221 in 5 of 63 ovarian cancer tissues and six cell lines was observed, including A2780, OVCAR3, SKOV3, and 3AO5 [60]. An in vitro cell viability assay showed that downregulation of miR-221/222 sensitized A2780/CP cells to cisplatin-induced cytotoxicity [58]. Another identified oncomir shown to promote proliferation of SKOV3, Hey, and OVCAR3 cells is miR-520b, which targets the ring finger protein 216 (RNF216) gene to promote cell growth. The negative correlation between miR-520b and RNF216 may present a new strategy for ovarian cancer [62]. In addition, numerous studies have shown that oncomirs play an important role in the acquisition of the ability to invade and form metastases by cancer cells. Overexpression of miR-10b in ovarian cancer has been reported in association with reduced amounts of transcription factor, HOXD10, in altered cells, leading to an increase in the levels of ras homolog family member C (RhoC) and matrix metallopeptidase 14 (MMP14), which are responsible for metastasis [64].

## 4. MiRNA Functions in Cancer Based on Regulation of DDR

**Figure 2:**
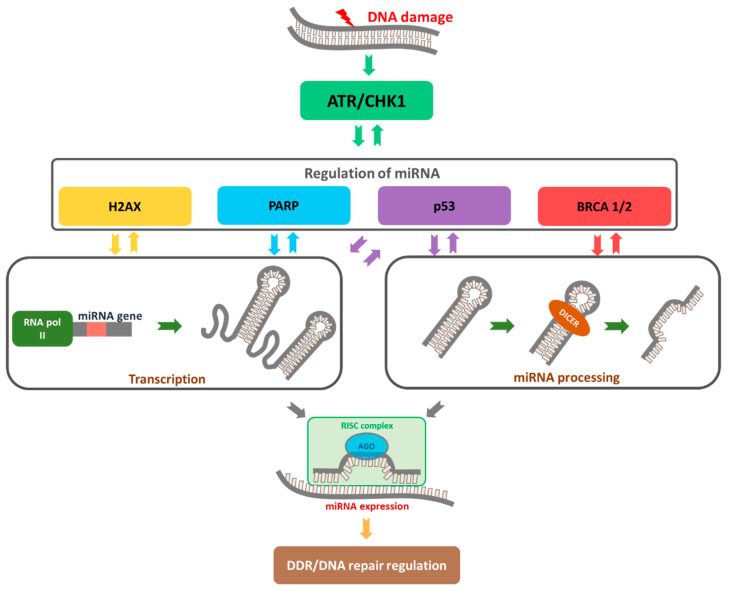
The role of miRNAs as a regulator of DDR. DNA damage triggers the activation of the ATR/CHK1 pathway, which is responsible for substrates phosphorylation (i.e., H2AX, p53, and BRCA 1/2) to repair DNA damage. Single-strand DNA damage is identified and repaired by poly (ADP-ribose) polymerase (PARP) pathway activation, predominantly through base excision repair (BER). With continuous PARP inhibition, ssDNA breaks are converted to dsDNA breaks during DNA replication in which replication forks stall at the point of DNA damage. Over-expressed or down-regulated miRNAs may affect DDR by changing the expression of repair genes.

The DNA damage response is a complex network involving proteins that are activated to facilitate detection of DNA damage and determine the survival or death of cells exposed to stress via stimulation of the signal transduction cascade [91]. Activation of the DDR pathway triggers cell cycle checkpoint activation and dividing alternation, in turn preventing the transfer of damaged DNA to daughter cells. Simultaneously, DNA repair mechanisms are activated. Upon repair of damage, cell cycle and division resume, allowing survival and continuation of function. If repair is not possible due to an excessive number of lesions, cells are eliminated by triggering programmed cell death or cellular aging, irreversible cell cycle arrest, and division processes [92], as presented in Figure 2. DDR modulates miRNA expression in transcriptional and post-transcriptional levels and involves miRNA degradation [66,93,94]. On the other hand, miRNAs may directly modulate the expression of multiple proteins in the DDR pathways. 

ATR and Ataxia telangiectasia mutated (ATM) kinases belonging to the phosphatidylinositol 3-kinase-related kinases (PIKK) family play central roles in activation of the DDR pathway [95]. Histone H2AX, one of the first known substrates for ATR and ATM kinases, is expressed in high-grade SOC, mucinous adenocarcinomas, and clear cell carcinomas. Significant changes in the gene and protein levels of H2AX have been reported in OC, supporting its predictive value as a biomarker [96].

### 4.1. MiRNAs Are Involved in Cell Cycle Disruption

**Figure 3:**
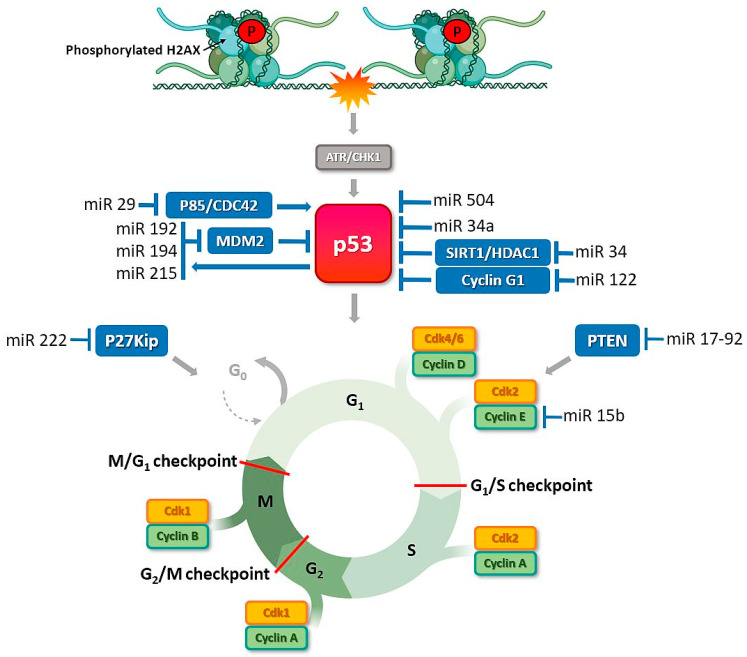
P53-induced miRNAs control cell cycle and cell survival in ovarian cancer. DNA damage stimulates ATR or DNA-PK kinases that activate TP53 to directly induce many kinds of miRNAs that repress cell-cycle regulators or allow to DNA repair. ATR transduces the DDR signal by phosphorylation of the checkpoint kinase CHK1, which results in cell cycle arrest and DNA repair. MiRNAs, e.g., miR-34a, a direct transcriptional target of TP53, participate in the regulation of TP53 activity. Activated TP53 translocates into the nucleus where it induces the transcription of several targets involved in cell cycle control, DNA repair, or apoptosis. DDR-related proteins that are MiRNAs recognizable are shown in blue box.

Imbalances in activities of miRNA molecules significantly affect cell cycle regulation, leading to excessive proliferation. Disruption of this process is often associated with direct interactions of miRNAs with key regulatory molecules of signaling pathways underlying proliferation, e.g., PTEN, Myc, Ras, and V-abl Abelson murine leukemia viral oncogene homolog 1 (ABL1), as well as proteins from the Rb pathway, cyclin-CDK complexes, or cell cycle inhibitors from families of inhibitors of CDK4 (INK4) and CDK-interacting protein/kinase inhibitory protein (Cip/Kip) [97]. Examples include miR-21, which is overexpressed in breast, ovary, and liver cancer, and a group of miR-17-92 members that inhibit PTEN phosphatase activity. Suppression of the gene encoding PTEN promotes cell proliferation. Another miRNA that influences the cell cycle is miR-15b, Figure 3. Decreased expression of miR-15b leads to an increase in cellular cyclin E1, resulting in lack of control during the transition from G1 to S phase. Ectopic expression of miR-192/215 induces cell cycle arrest and targets a number of transcripts that regulate G1/S and G2/M checkpoints [98,99]. These miRNAs are transcriptional targets of p53 and also upregulate p53 by downregulating Murine Double Minute gene 2 protein (MDM2) [100].

### 4.2. Functional miRNAs in Activation of the “Response Track” to DNA Damage and the Role of H2AX Histone

**Figure 4:**
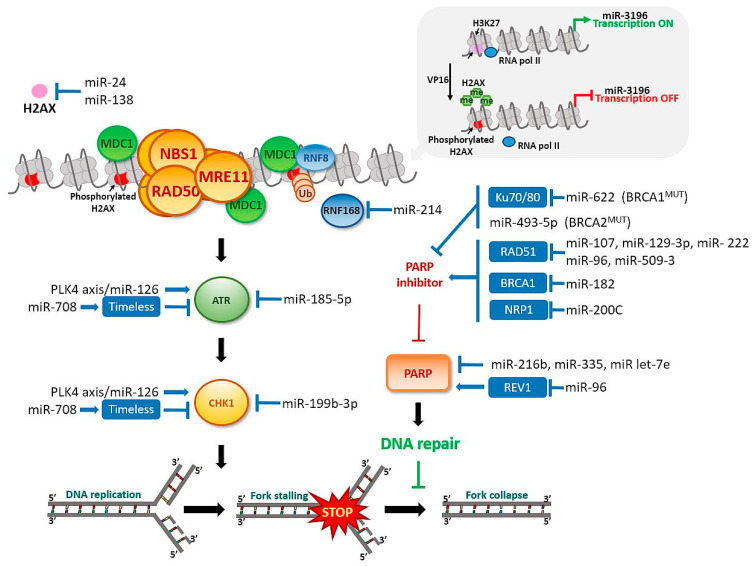
MiRNAs’ involvement in the DNA damage response in ovarian cancer. DNA synthesis inhibition or damage induces checkpoint responses. PARP and checkpoint proteins controlled by the ATR–CHK1 pathway prevent fork collapse, replication stress, and genome instability. DDR-related proteins that are MiRNAs recognizable are shown in blue box.

In response to DNA damage, H2AX is phosphorylated by DNA-dependent protein kinase, catalytic subunit (DNA-PKc), which is also a member of the PIKK family. The histone is phosphorylated at serine 139 (known as γH2AX) and initiates attachment of subsequent elements of the signaling pathway [101]. At the same time, histone H2AX is dephosphorylated at tyrosine 142 and constitutively phosphorylated under conditions of no DNA damage [102]. Dephosphorylation promotes direct attachment of the mediator of DNA damage checkpoint protein 1 (MDC1) protein to γH2AX. Anchoring of MDC1 at the site of damage is a platform for activation of other proteins belonging to the DDR pathway and the MRN (MRE11, Rad50, NBS1)/ATM complex. This enhances local ATM kinase activity and extension of the H2AX phosphorylation region to include nucleosomes adjacent to DNA damage [103,104]. The clusters favor extensive formation of γH2AX, which plays an important role in accumulation and maintenance of components of the DDR pathway, such as MRN, and proteins related to DNA repair, including BRCA1 and p53-binding protein 1 (53BP1). Binding of phosphorylated MDC1 to γH2AX facilitates attachment of E3, RNF8 (E3 ubiquitin-protein ligase), and RNF168 ubiquitin ligases to the lesion site, which promote association of BRCA1 and 53BP1 via ubiquitination of chromatin [91,105,106,107,108]. Downregulation of ubiquitin ligase RNF8, which is necessary for γH2AX to recruit DNA repair proteins to DNA damage sites, via miR-214, induces chromosomal instability in ovarian cancer [109], Figure 4. Thus, H2AX histone appears to play a pivotal role as an early indicator protein for DDR. Previous reports showed that miR-24 and miR-138 regulate H2AX via 3’-UTR attachment. Overexpression of miR-138 inhibited homologous recombination and enhanced cellular sensitivity to multiple DNA damage agents (cisplatin, camptothecin, and ionizing radiation) [98]. MiR-138 was recently identified as an effective tumor suppressor in multiple malignancies including ovarian cancer [56]. MiR-24 mediates suppression of H2AX in terminally differentiated blood cells, which renders them hypersensitive to gamma-irradiation, deficient in DSB repair, and susceptible to chromosomal instability [110]. Another study reported that overexpression of miR-24-insensitive CHEK1 does not rescue the DNA repair phenotype induced by miR-24 [111]. Moreover, γH2AX has been shown to regulate miR-3196 gene expression. H3K27 trimethylation in the miR-3196 promoter region regulated via H2AX phosphorylation at Ser139 is a key step in H2AX-mediated apoptosis [112]. Furthermore, Fra-1 transcriptional factor and miR-134 are upregulated in ovarian cancer tissues. MiR-134 enhances H2AX S139 phosphorylation via activation of c-Jun NH2 kinase (JNK) and promotes DNA repair through non-homologous end-joining (NHEJ) [113].

### 4.3. MiRNAs Contributes to DSB DNA Damage Repair System

The primary function of the DDR pathway is to identify DNA damage and, where possible, initiate repair processes. The majority of DNA damage is repaired by the triggering of catalytic event sequences involving multiple proteins, including base excision repair (BER), NER, mismatch repair (MMR), HR, and NHEJ. Two types of nucleotide excision repair pathways exist. One is active during transcription (transcription coupled repair, TCR), while the other is independent of transcription (Global Genomic Repair, GGR) [114,115]. Activation of a specific mechanism depends on the type of DNA damage. BER, NER, and MMR pathways play key roles in repairing damage such as single DNA strand breaks (SSB), replication errors, insertions, deletions, and adducts [116,117].

Double-strand breaks (DSB) are one of the most dangerous types of DNA damage, and a single unrepaired DSB is sufficient to trigger apoptosis [118]. Two processes are involved in repair of double-strand breaks, specifically, HR and NHEJ. Homologous recombination can occur in the S and G2 phases of the cell cycle. On the other hand, repair of damage by non-homologous recombination is possible at any phase of the cell cycle, including G0 [119,120,121]. In HR repair, proteins of the MRN complex and BRCA1 C-terminal Interacting Protein (CtIP) play a key role. These proteins are involved in formation of short sections of single-stranded DNA (ssDNA), which initiate repair of damage through homologous recombination. With the aid of BRCA1, BRCA2, and RAD51 proteins, short sections of single-stranded DNA are joined to the undamaged template. In conjunction with the activities of polymerase, nuclease, helicase, and other proteins, damage is repaired. HR is also involved in resumption of replication caused by blockage of replication forks [122,123]. One of the key proteins of the MRN complex is RAD51. In an earlier study, upregulation of miR-210 significantly suppressed expression of RAD51, while upregulation of miR-373 inhibited RAD52 (which recognizes double-strand breaks and adheres to the free ends of the break) [110]. Another study by Moskwa et al. [124] consistently showed that miR-182 downregulates BRCA1 expression. MiR-182 enhances BRCA1 protein levels and protects against irradiation-induced cell death, while its overexpression reduces BRCA1 protein, impairs homologous recombination-mediated repair, and renders cells hypersensitive to irradiation. Subsequently, ability of HR to stimulate DSB repair is significantly decreased [124].

In the case of NHEJ, DSB are recognized by the heterodimeric Ku70/Ku80 protein complex, which binds DNA-PKc kinase. Subsequently, DNA polymerases and DNA ligase IV, enzymes that process DNA ends, are recruited and activated. In addition, it is possible to repair DNA damage related to joining non-homologous ends. This process, known as alternative NHEJ (alt-NHEJ) or microhomological-mediated end joining (MMEJ), occurs independently of the Ku protein [125,126]. Earlier literature suggests that miR-101 is able to successfully regulate DNA-PKcs and ATM through attaching to their 3’-UTRs. Specifically, upregulation of miR-101 significantly reduced the protein levels of DNA-PKcs and ATM in tumor cells and sensitized them to radiation, both in vitro and in vivo. Thus, miR-101 is a potential option for use in DNA DSB repair gene targeting to optimize the effects of tumor radiotherapy [127].

### 4.4. MiRNAs Modulate Activity of p53, a Key Protein of the DDR Pathway

The p53 protein is a key suppressor of neoplastic transformation that regulates transcription of numerous genes and interacts directly with multiple proteins. p53 is implicated in a number of critical cell processes, including DNA repair, cell cycle, and programmed cell death. Under conditions where the cell is not exposed to stress factors, the p53 protein level is relatively low. This may be attributed to interactions with (MDM2), which blocks transcriptional activity of p53, leading to its ubiquitination-dependent degradation. MDM2 synthesis is regulated by p53, generating a negative feedback loop leading to a decrease in p53 levels after induction. The imbalance between p53 and MDM2 levels is a critical step in p53 activation [128] and occurs when activated ATM and/or ATR kinase phosphorylates the p53 protein at serine 15 and CHK2 at serine 20. ATM also phosphorylates MDM2 in response to DNA damaging agents. As a result of these modifications, interactions of MDM2 with p53 are blocked, leading to the inhibition of MDM2-dependent degradation and, consequently, accumulation of p53. Thus p53 is activated as a result of post-translational modifications, such as phosphorylation, acetylation, methylation, and ubiquitination. The p53 protein serves as a transcriptional factor to regulate expression of target genes, which also occurs through recruitment of coactivators or corepressors. Among these molecules, acetyltransferases are known to play an important role. Enzymes such as CREB-binding protein (CBP), p300, Tip60, human males absent on the first (Hmof), or P300/CBP-associated factor (PCAF) acetylate p53 and histones alter chromatin conformation, increasing the availability of the DNA template for transcription machinery. In response to DNA damage, CBP and/or p300 acetylate p53 at six lysine residues, which present a target for MDM2 ubiquitination, thereby increasing the stability of p53 and binding ability to DNA [129]. 

Depending on the type and extent of DNA damage, various post-translational modifications of p53 are initiated, which translate into different cellular responses. Thus, p53 serves as the main decision switch for survival or death. Several groups can be distinguished among the genes regulated by p53 in response to DNA damage. One of these categories is negative regulators/inhibitors of the cell cycle, such as p21, 14-3-3σ, and GADD45α, which trigger cell cycle arrest and division, facilitating repair of DNA damage [130]. In response to DNA damage, p53 is involved in the regulation of processes related to cell metabolism and autophagy. In addition, transcription-independent and miRNA-dependent p53 functions have been reported. MiRNAs either directly target the 3′ UTR of p53 or indirectly regulate p53 activity by modulating proteins associated with p53. Among these microRNAs, miR-504 negatively regulates p53 expression through binding two DNA *cis* elements located in the 3′ UTR region [131]. DNA damage promotes the p53-dependent upregulation of miR-192, miR-194, and miR-215. Studies also have revealed the existence of a specific p53 binding site around the miR-194/miR-215 cluster [132].

In addition to direct binding to p53, several miRNAs, including miR-34a, miR-29, and miR-122, indirectly modify p53 activity [133,134,135,136]. MiR-34a is a direct transcriptional target of p53 [137,138,139], whereby p53 upregulates miR-34a expression via binding to specific promoter regions. MiR-34a positively regulates p53-dependent apoptosis through another SIRT1 [133]. MiR-34a expression is low in patients with chromosomal abnormalities involving the tumor protein p53 (TP53) gene locus and is associated with poorer prognosis and shorter survival. Mutations or deletions in the 17p13 region of the TP53 gene locus may indirectly lead to reduced miR-34a expression [140]. Another miRNA family involved in p53 regulation is miR-29. Members of this family directly suppress phosphoinositide 3-kinase subunit (P85a) and cell division control protein 42 homolog (CDC42), both of which negatively regulate p53. As a result, miR-29 positively upregulates the p53 level and induces apoptosis and DNA repair in a p53-dependent manner [134].

## 5. MiRNAs Associated with DNA Repair Checkpoint Proteins: New Options for Optimizing Ovarian Cancer Therapy

### 5.1. PARP

PARP is an important protein involved in the repair of single-stranded DNA breaks, seen in Figure 3. PARPis have been shown to selectively kill cells with defective HR pathways as a result of synthetic lethality [141]. However, a large proportion of HR-mutated cancers gain resistance to these therapeutic agents. PARPi sensitivity is modulated through downregulation of critical DNA repair genes as a consequence of alterations in miRNA profiles. PARPi resistance may be promoted by miR-622 that modulates the balance of DNA repair through selective inhibition of expression of NHEJ proteins, such as KU70/80, which maintain genome stabilization after treatment with DNA-damaging agents or PARPi. High expression of miR-622 in BRCA1^MUT^ epithelial ovarian cancer is associated with prediction of poorer disease-free and overall survival [69]. The functional impact of miR-493-5p has been characterized in BRCA2^MUT^ cancer cells. MiR-493-5p induces platinum and PARPi resistance by affecting several pathways, including single-strand annealing (SSA), R-loops, and replication fork stability [142]. In contrast, miR-107, miR-129-3p, and miR-222 increase sensitivity to PARP inhibitors and ionizing radiation by causing a reduction in the DNA damage response via impairing the HR pathway based on targeting of RAD51 [143]. Mi182 exerts similar effects and enhances PARPi sensitivity by downregulating BRCA1 [124]. Moreover, expression of miR-96 is increased in many cancer types. This miRNA enhances sensitivity to platinum agents and PARP via downregulation of the DNA repair proteins REV1 and RAD51 [144]. Another study on a patient-derived xenograft (PDX) model of high-grade serous ovarian carcinoma (HGSOC) revealed an essential role of miR-509-3 in tumor suppression and HR signaling, along with increased sensitivity to PARPi treatment [59]. Furthermore, PARP1 expression could be altered by miR-335 or miR-216-b. MiR-335 plays a dual role as either a tumor promoter or suppressor in a wide variety of cancers. However, expression is reduced in ovarian cancer cells and miR-335 shown to effectively increase sensitivity to cisplatin treatment [61]. MiR-216-b regulates apoptosis and autophagy and directly binds to PARP1 mRNA, leading to inhibition of its expression. Lower expression of miR-216b is reported in cisplatin-resistant ovarian cancer cells [145]. Recent studies have additionally demonstrated a role of Neuropilin 1 (NRP1) in response to ovarian cancer therapies. NRP1 is expressed at high levels in resistant cells (SKOV3) and shown to be upregulated in partially sensitive cells (UWB-BRCA) upon prolonged olaparib treatment, resulting in poor drug response. MiR-200c targets and suppresses NRP1 expression in OC cells resistant to therapy, leading to the restoration of olaparib sensitivity [89].

Platinum-resistant ovarian tumors display low miR-Let7i expression. Conversely, its gain of function results in restoration of drug sensitivity in chemoresistant ovarian cancer cells [146]. Agomir is a type of specially labelled and chemically modified double-stranded microRNA that can regulate the biological functions of target genes by mimicking endogenous microRNAs. Let-7e agomir suppressed the mRNA levels of PARP1 and insulin-like growth factor I (IGF-1) while its downregulation enhanced PARP1 and IGF-1 expression [37]. Specific miRNA expression profiles could therefore serve as biomarkers in ovarian cancer to predict response to PARPi therapy. 

### 5.2. ATR

The ATR protein belonging to the phosphatidylinositol 3-kinase-related (PI3K) family is involved in the signaling of stalled replication forks and maintaining genomic stability during the S phase, along with its partners ATR interacting protein (ATRIP) and replication protein A (RPA) [147]. A broad spectrum of DNA damage, such as single- and double-stranded DNA breaks, cross-links, and adducts, can lead to the activation of ATR [148]. ATR is referred to as the “master of DDR”, highlighting the relevance of miRNAs implicated in DDR pathways as novel therapeutic targets for ovarian cancer. MiR-383-5p and miR-185-5p have been shown to be associated with ATR kinase. MiRNA-383-5p is predominantly downregulated and acts as a tumor suppressor in several human cancer types, such as gastric, glioma, medulloblastoma, and testicular embryonal carcinomas. In the mammalian ovary, miR-383 plays a functional role in follicle development [63]. MiR-185 suppresses expression of ATR and activation of its downstream effector, CHK1, which are induced by ionizing radiation. Furthermore, miR-185 is reported to induce G1 cell cycle arrest and apoptosis, inhibiting cancer cell proliferation [149].

A serine/threonine-protein kinase, PLK-4, has been identified as a target of miR-126, which is downregulated in various cancers in correlation with tumor progression and poor prognosis. Earlier experiments showed that PLK-4 knockdown led to a decrease in expression of ATR and CHK1, supporting its interactions with the ATR/CHK1 pathway. Moreover, changes in miR-126 expression led to PLK-4, ATR, and CHK1 dysregulation [67]. Based on these findings, it is proposed that miR-126 inhibits cancer progression via regulation of the cell cycle through inducing alterations in the ATR/CHK1 pathway.

MiR-708 overexpression is associated with suppression of the ATR/CHK1 pathway. Timeless was a direct target of miR-708. Total and phosphorylated ATR and CHK1 levels were decreased in cells overexpressing miR-708 after cisplatin treatment [68], supporting the utility of this miRNA as a potential therapeutic target. Overall, the effects of miRNAs on ATR kinase levels signify their potential application as new therapeutic targets for ovarian cancer.

### 5.3. CHK1

Checkpoint kinase 1 is a serine/threonine kinase encoded by the CHEK1 gene activated in response to DNA damage and replication stress that is proposed to regulate mitotic progression [150]. ATR and CHK1 share the same signaling pathway. However, in addition to ATR-induced activation, CHK1 can be autophosphorylated and activated independently of ATR [151].

Numerous studies have validated the oncogenic association of miR-424. Decreased expression of miR-424-5p is significantly associated with distant metastasis in high-stage (stage III and IV) ovarian cancers [72]. Moreover, downregulation of miR-424 contributes to the progression of cervical cancer via upregulation of target CHEK1 gene expression and phosphorylation of CHK1 protein, while its overexpression inhibits CHK1 expression [152]. 

Another miRNA downregulated in serous ovarian tumours is miR-195-5p [153]. In lung tumor tissues, miR-195 expression is low and associated with poor survival outcomes, while overexpression of miR-195 results in suppression of cancer cell growth, migration, and invasion. CHK1 has been identified as a direct target of miR-195. Low expression of miR-195 leads to high expression of CHK1, which is associated with poor prognosis in patients with lung tumors [154]. 

Expression of miR-330-5p regulates the development of different tumor cell types. In cutaneous malignant melanoma, miR-330 suppresses cell proliferation as well as expression of tyrosinase and protein disulfide-isomerase A3 (PDIA3) [155]. Conversely, its overexpression could promote apoptosis of prostate cancer cells through E2F1-mediated suppression of RAC-alpha serine/threonine-protein kinase (Akt) phosphorylation [156]. In esophageal adenocarcinoma, miR-330 was shown to modulate neoadjuvant chemoradiotherapy sensitivity [157], while in non-small cell lung cancer, its overexpression inhibited NIN1/RPN12 binding protein 1 homolog (NOB1) expression and cancer cell growth [158]. On the other hand, downregulation of miR-330-5p is reported in epithelial ovarian cancer tissues [159]. Moreover, the long non-coding RNA LINC01224 modulates expression of miR-330-5p, resulting in the downregulation of CHEK1 in hepatocellular carcinoma [160]. CHEK1 has also been identified as a direct target of miR-497, whereby expression of CHK1 protein is negatively regulated by miR-497 and upregulated under conditions of downregulation of miR-497 [161]. Other miRNAs responsible for suppressing expression of CHK1 and Wee1 are miR-16 and miR-26a. During genotoxic stress, p53 upregulates miR-16 and miR-26a, in turn attenuating expression of Wee1 and CHK1 [29]. These effects promote accumulation of cells in the G1 phase and, consequently, apoptosis. Additionally, miR-199b-3p overexpression in ovarian cancer suppresses E-box binding homeobox (ZEB)1 and CHK1. Moreover, E-cadherin and EMT expression were increased, which led to the conclusion that miRNA-199b-3p may suppress the progression of ovarian cancer via the CHK1/E-cadherin/EMT signaling pathway [162].

The collective findings highlight the significance of CHK1 as a key pharmacological target. Inhibition of CHK1 protein induces sensitization of cancer cells to genotoxic therapy and is recognized as beneficial in the treatment of ovarian cancer [163]. Thus, downregulation of CHK1 through targeted miRNAs may present an effective novel therapeutic strategy. 

## 6. Conclusions

**Figure 5:**
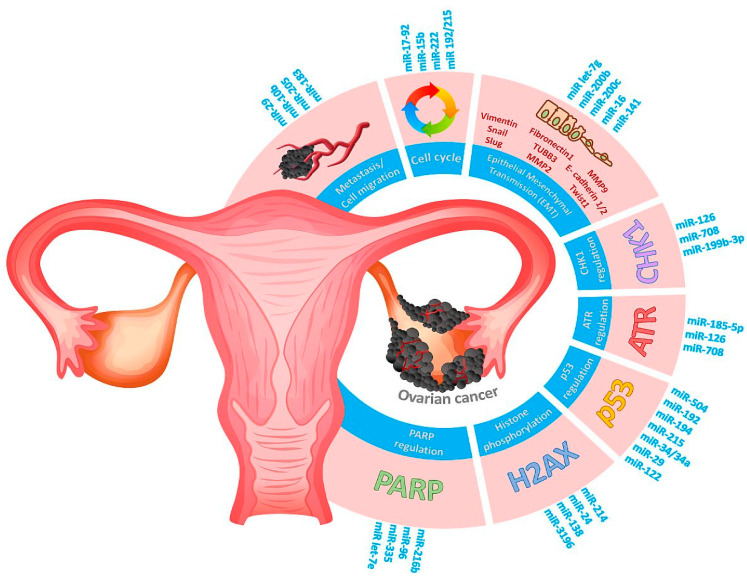
Schematic diagram representing several hallmarks of cancer contributing to pathogenesis of ovarian cancer. RNA markers involved in DDR signaling (PARP1, histone H2AX, P53, ATR, CHK1 regulation), cell cycle disturbance, metastasis, or epithelial–mesenchymal transmission are highlighted in blue.

Since early detection tools are lacking, ovarian cancer is often diagnosed at late stages, which substantially contributes to the high mortality rates. MiRNAs are implicated in regulating almost every aspect of the DDR, DNA repair, and cell cycle arrest (Figure 2, Figure 3, Figure 4 and Figure 5). MiRNAs may be an alternative method to identify DDR defects in patient therapy. Previously, a miRNA-score was developed that was associated with genome instability and predicted the outcome of ovarian cancer based on mutations in caretaker genes. The authors described 10 miRNAs. Six of them had higher expression than the median value across the dataset and were associated with a high frequency of mutation (miR-151, miR-301b, miR-505, miR-324, miR-502, and miR-421). The other four (let-7a, miR-320, miR-146a, and miR-193a) had lower expression associated with a lower frequency of mutation in the cancer genome [164]. 

Improved understanding of the critical roles of miRNAs in DDR and chemotherapy may therefore provide novel insights with a view to expanding their application as potential tools, biomarkers, or sensitizers in cancer treatment. Promising for increasing the effectiveness of ovarian cancer treatment is the combined therapy with miRNA and chemotherapeutic agents. The role of miRNA in modulating the ovarian cancer cells’ sensitivity to chemotherapeutic agents in multidrug-resistance has been confirmed. It has been revealed that, e.g., decreased resistance to paclitaxel was associated with the upregulation of miR-29b, let-7i, miR-199a, miR-200a, miR-200c, and miR-215, while decreased resistance to platinum agents is related to the upregulation of miR-149, miR-155, miR152, miR-199a, miR200b, miR- 200c, miR-30d, miR-34c, miR-363, miR-497, miR-506, miR-9, and let-7i and to the downregulation of miR-23a and miR-603 [165]. Recent studies demonstrated also that miR-200c significantly enhanced the anticancer efficacy of olaparib in drug-resistant OC cells, which gives hope for optimizing the clinical use of PARPi [89]. Further research is warranted to clarify the correlations among miRNAs, DDR, and ovarian cancer. Continued advancements in miRNA research should allow clarification of the mechanisms’ underlying cancer development, individualization of treatment, and improvement in prognosis for patients with ovarian cancer.

## Figures and Tables

**Table 1:** Molecular functions of miRNAs in ovarian cancer.

MiRNAs as Oncogenes	Ref	MiRNAs as Suppressors	Ref
miR-138	[26,27]	miR-16	[28,29]
miR-200 a, a-3p, b, c	[30,31,32,33]	miR-10a, 10b	[34]
miR-141	[30,33]	miR-29	[35,36]
miR-429	[30,33]	miR-let-7	[37,38,39,40]
miR-205	[41]	miR-31, 31-5p	[42,43]
miR-126-3p	[44]	miR-506-3p	[45]
miR-183	[46]	miR-424-5p	[47]
miR-760	[48,49]	miR-503-5p	[47]
miR-151	[50]	miR-199a-5p	[51]
miR-21-5p	[52]	miR-34	[53]
miR-106a	[54]	miR-340-5p	[55]
miR-195	[54]	miR-138	[56]
miR-222	[57,58]	miR-509-3	[59]
miR-221	[57,58,60]	miR-335-5p	[61]
miR-520b	[62]	miR-383	[63]
miR-10b	[64]	miR-185	[65]
miR-21	[66]	miR-126	[67]
miR-17-92	[66]	miR-708	[68]
miR-622	[69]	miR-200c	[18,70,71]
miR-424-5p	[72]		

## Data Availability

The data presented in this study are available on request from the corresponding author.
